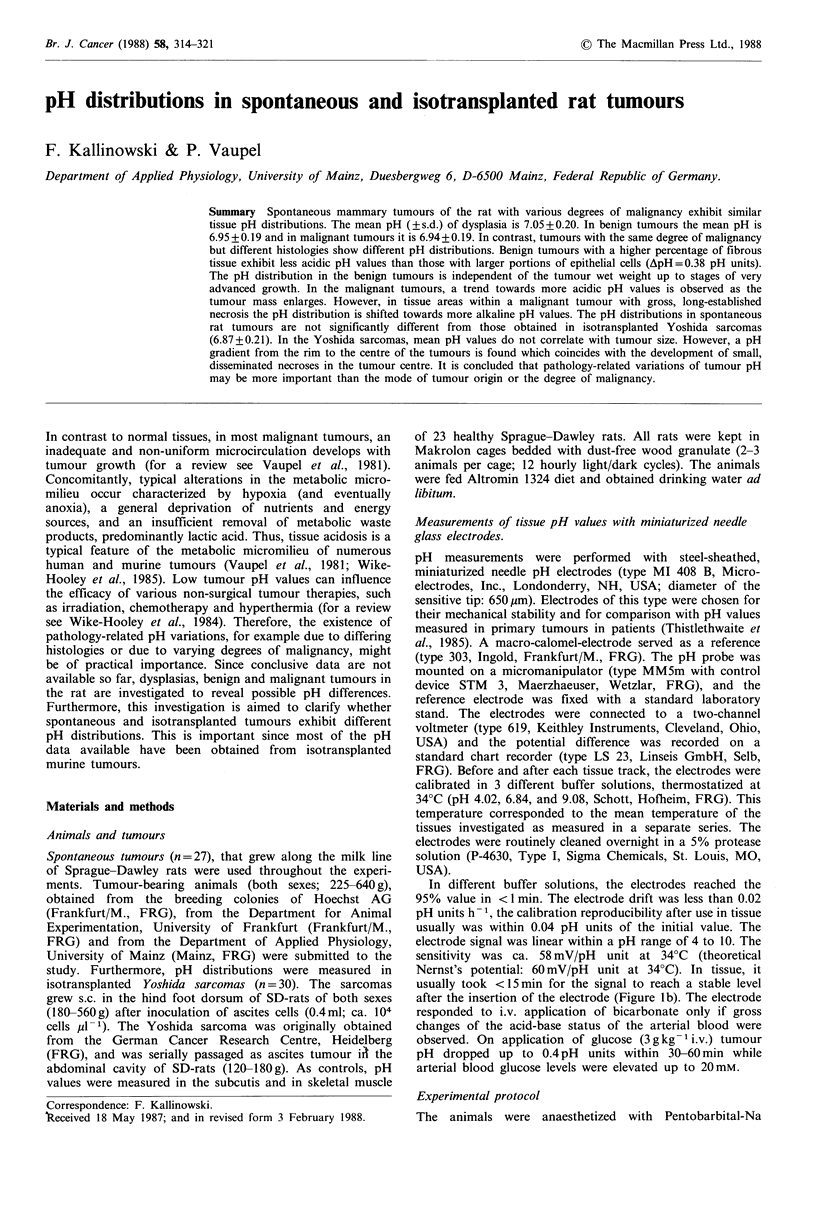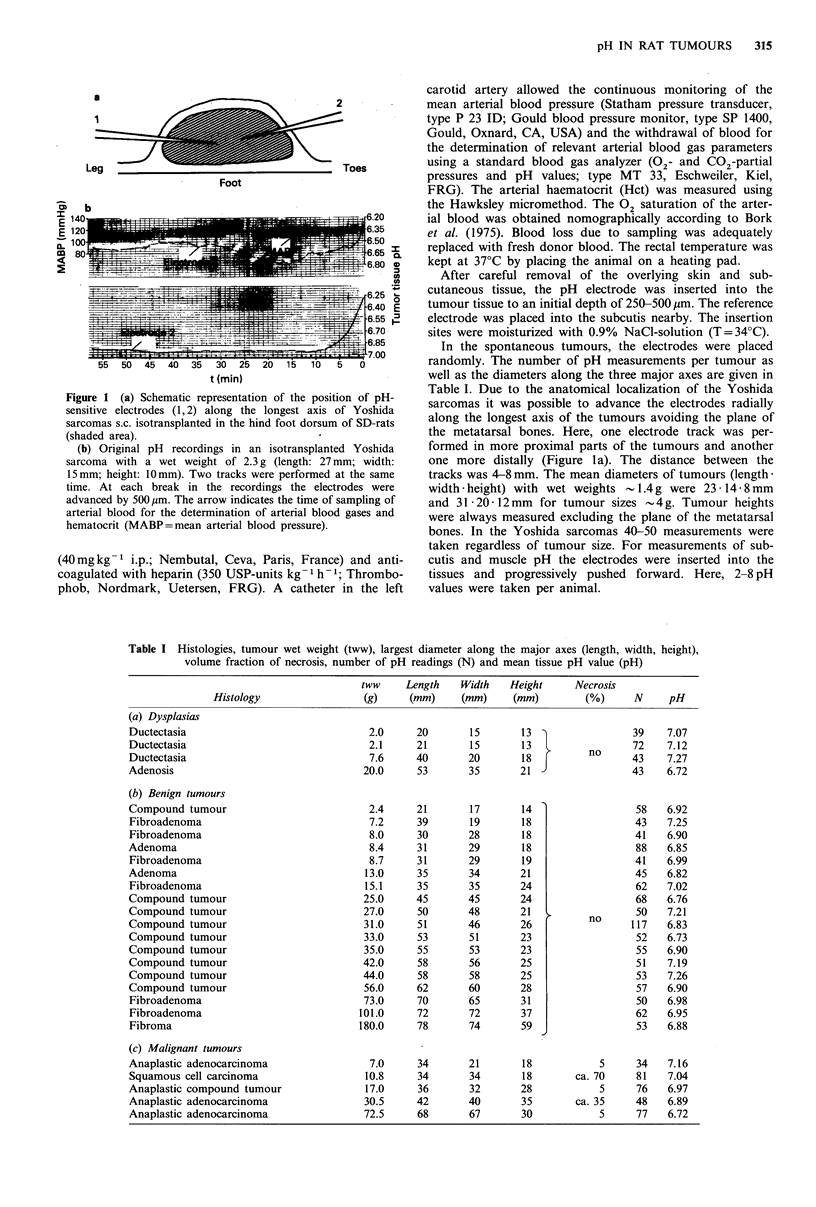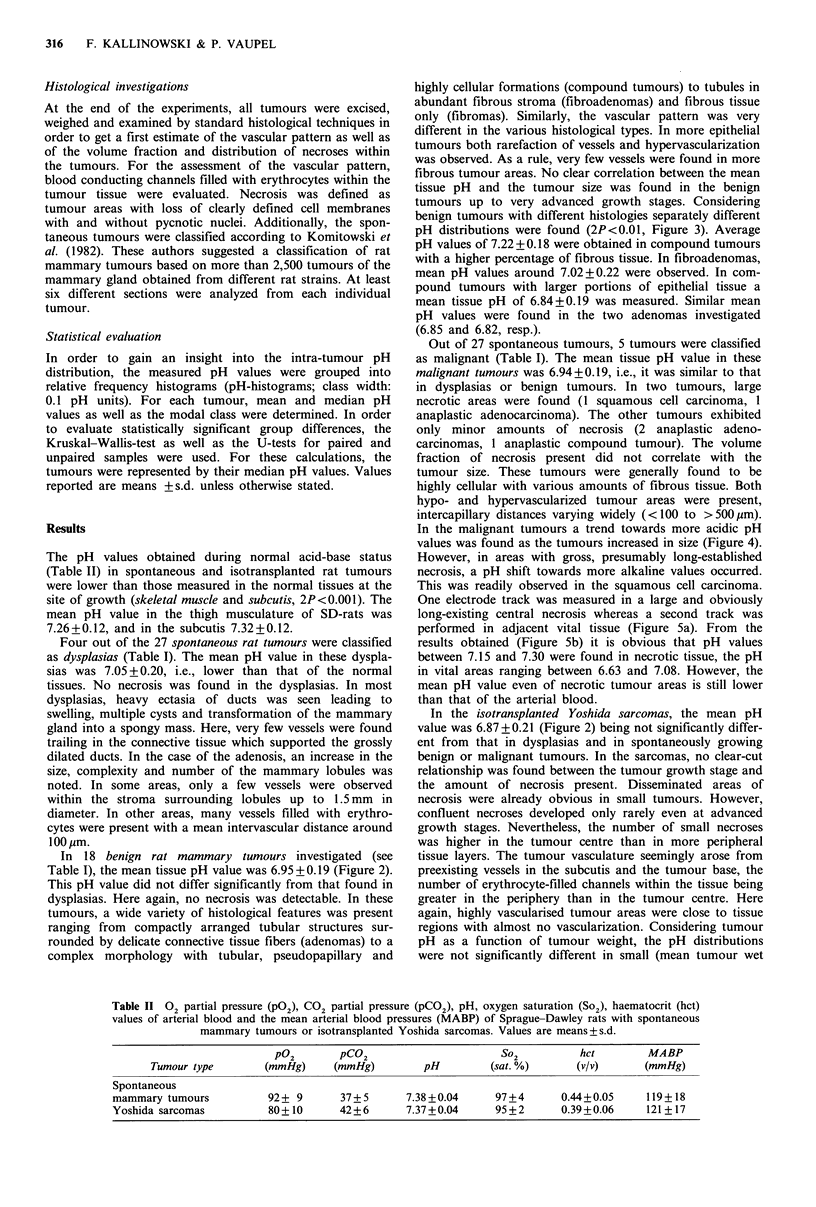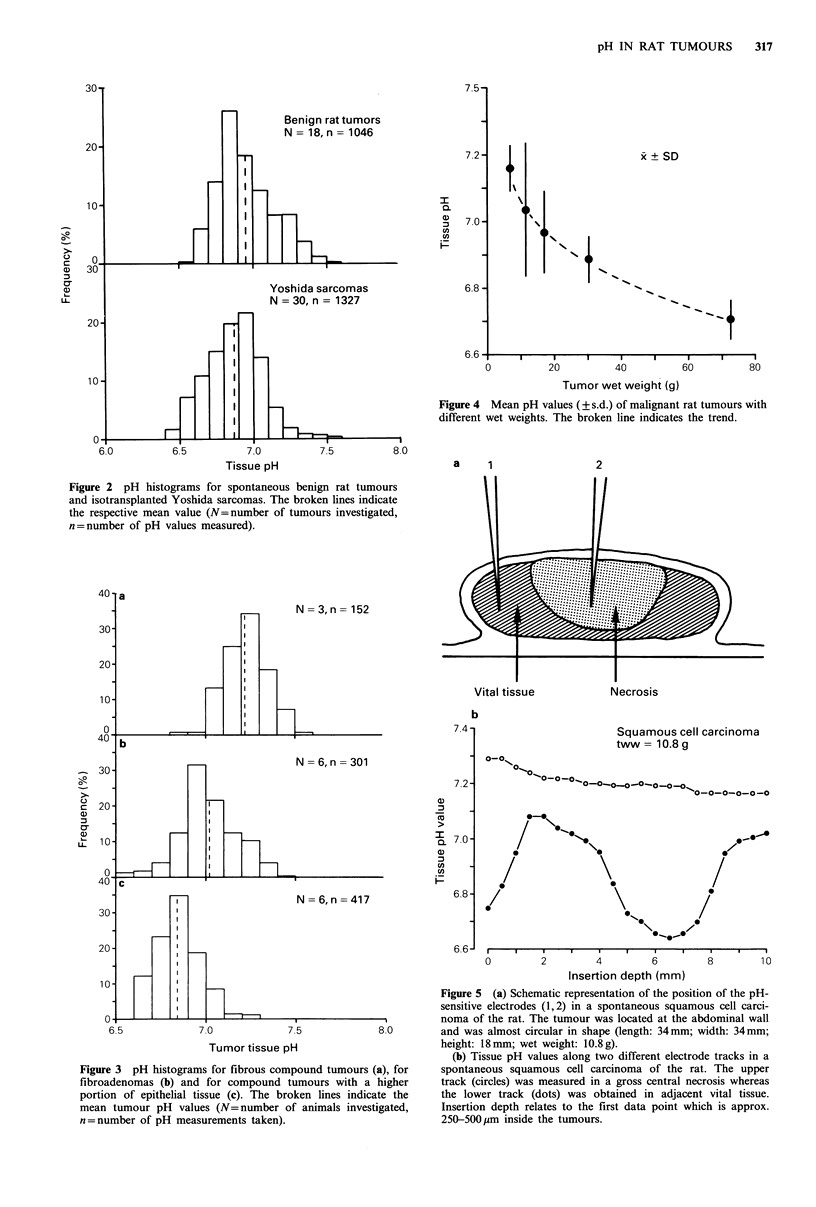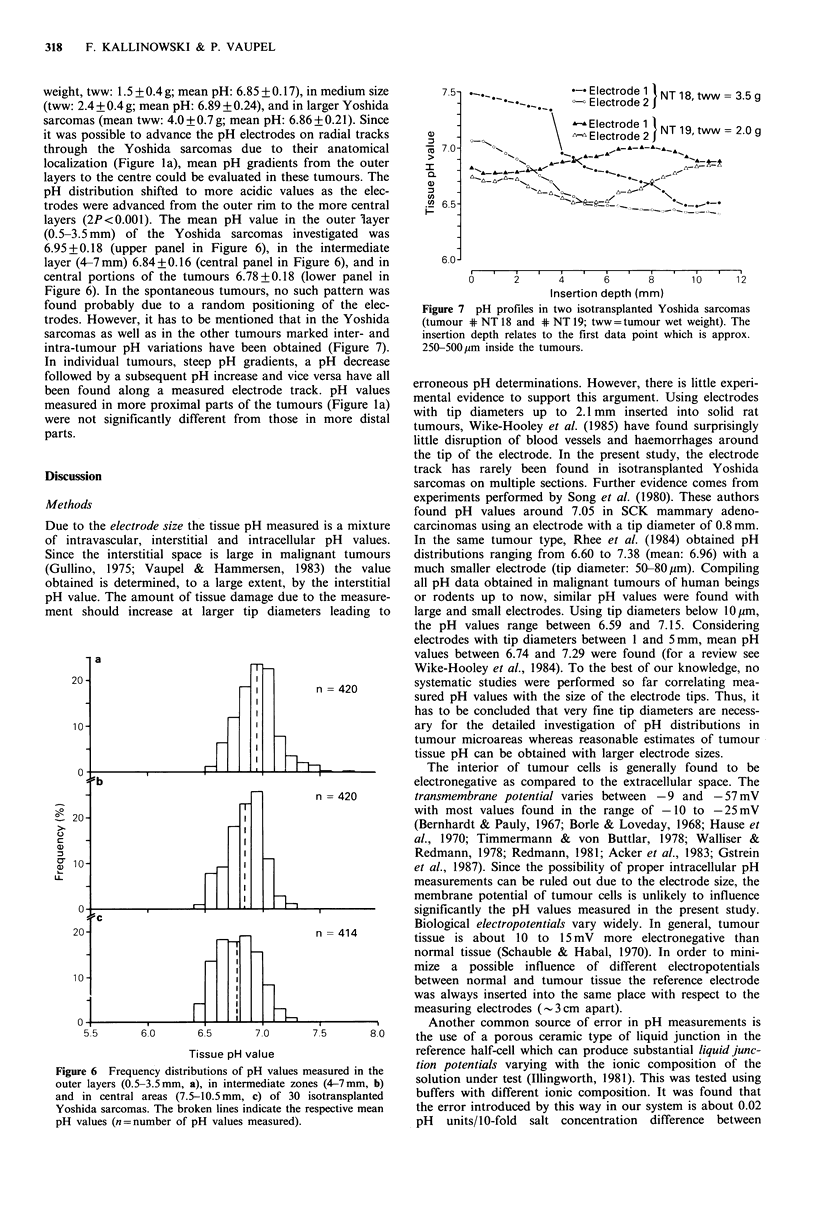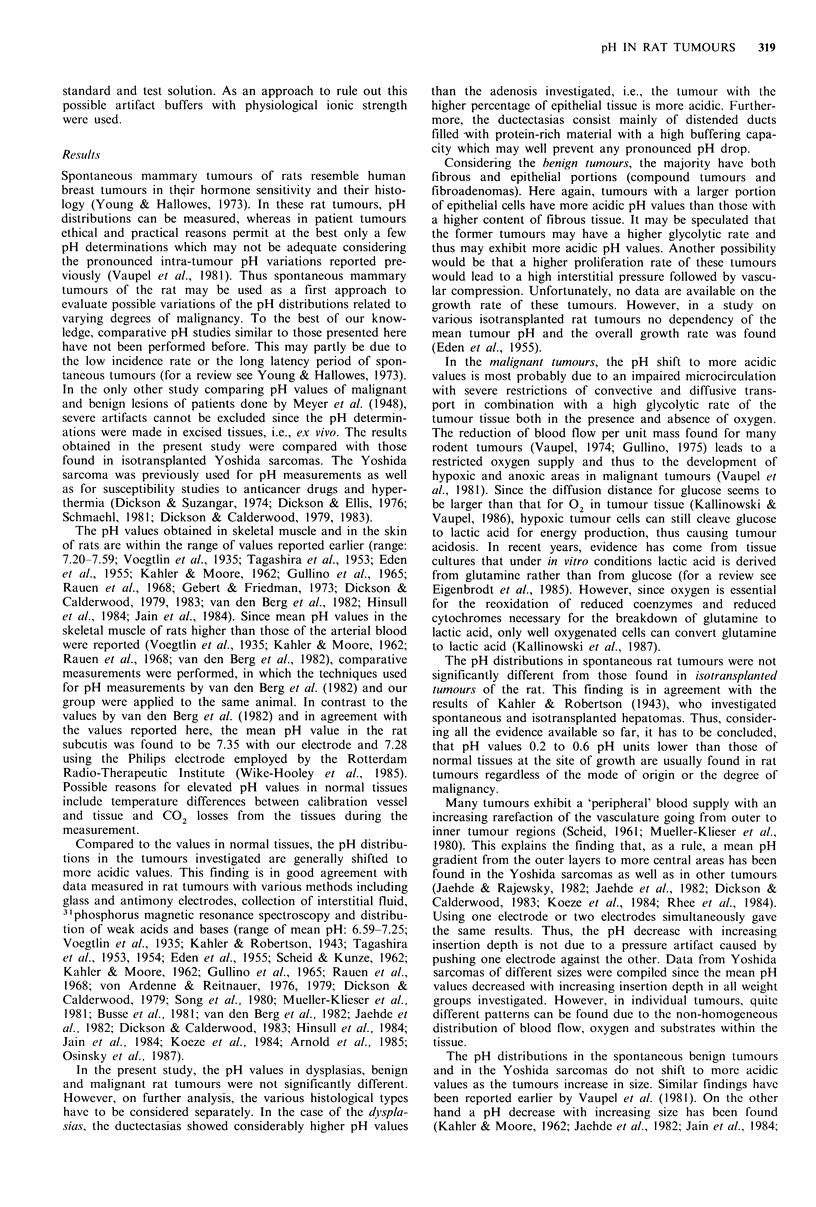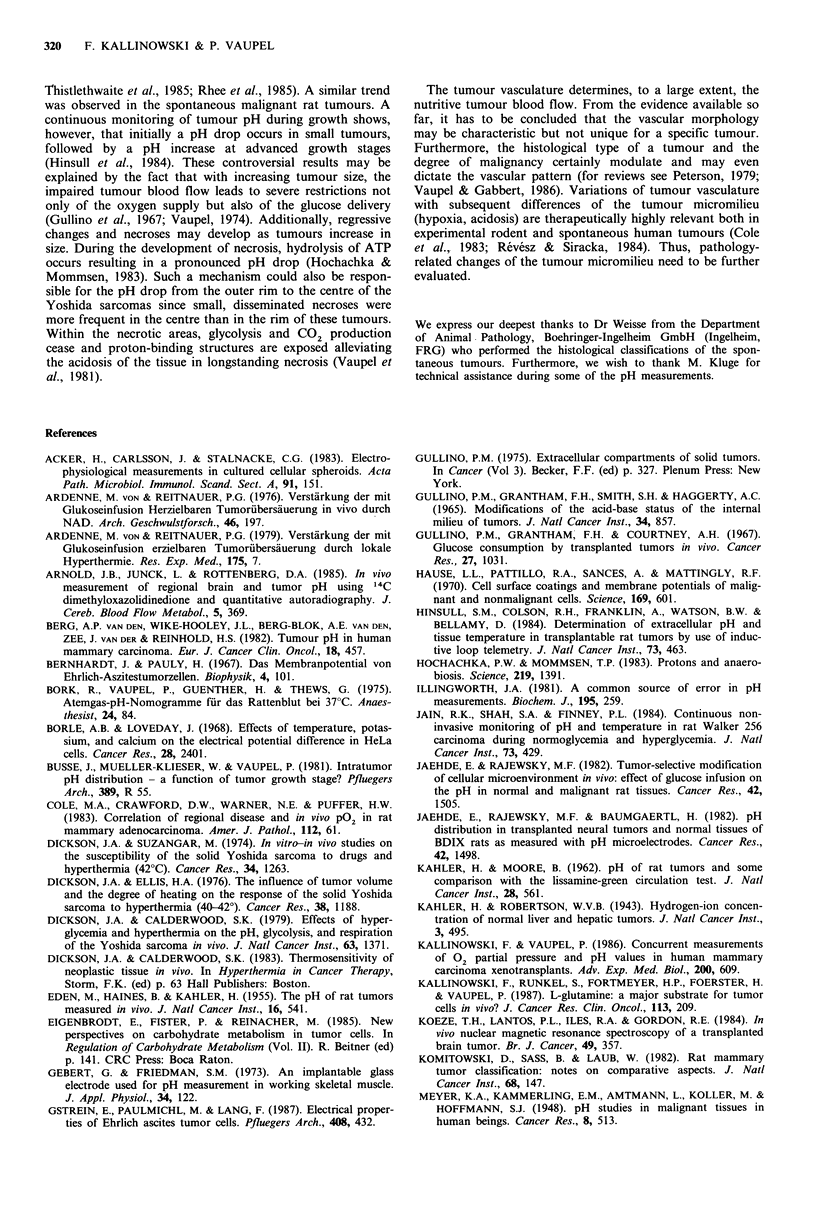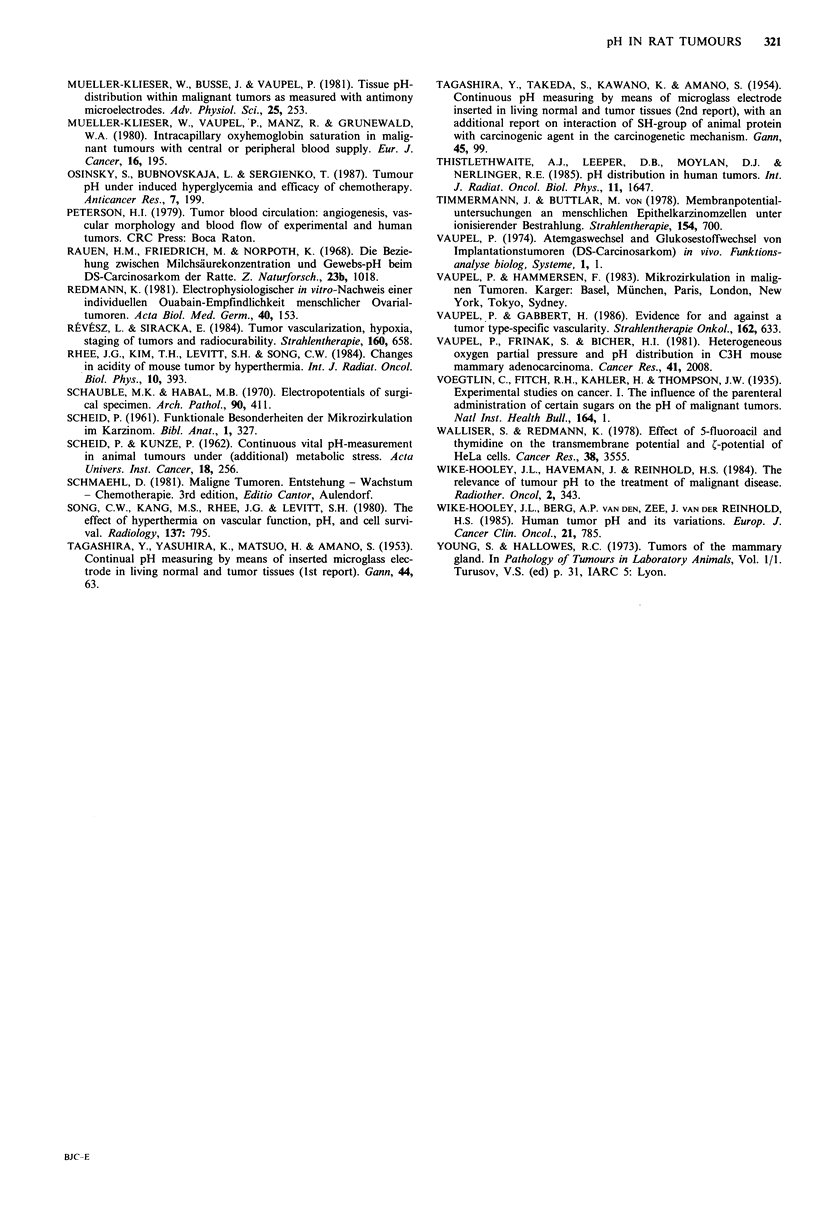# pH distributions in spontaneous and isotransplanted rat tumours.

**DOI:** 10.1038/bjc.1988.210

**Published:** 1988-09

**Authors:** F. Kallinowski, P. Vaupel

**Affiliations:** Department of Applied Physiology, University of Mainz, Federal Republic of Germany.

## Abstract

Spontaneous mammary tumours of the rat with various degrees of malignancy exhibit similar tissue pH distributions. The mean pH (+/- s.d.) of dysplasia is 7.05 +/- 0.20. In benign tumours the mean pH is 6.95 +/- 0.19 and in malignant tumours it is 6.94 +/- 0.19. In contrast, tumours with the same degree of malignancy but different histologies show different pH distributions. Benign tumours with a higher percentage of fibrous tissue exhibit less acidic pH values than those with larger portions of epithelial cells (delta pH = 0.38 pH units). The pH distribution in the benign tumours is independent of the tumour wet weight up to stages of very advanced growth. In the malignant tumours, a trend towards more acidic pH values is observed as the tumour mass enlarges. However, in tissue areas within a malignant tumour with gross, long-established necrosis the pH distribution is shifted towards more alkaline pH values. The pH distributions in spontaneous rat tumours are not significantly different from those obtained in isotransplanted Yoshida sarcomas (6.87 +/- 0.21). In the Yoshida sarcomas, mean pH values do not correlate with tumour size. However, a pH gradient from the rim to the centre of the tumours is found which coincides with the development of small, disseminated necroses in the tumour centre. It is concluded that pathology-related variations of tumour pH may be more important than the mode of tumour origin or the degree of malignancy.


*Br. J. Cancer* (1988) **58**, 314–321
© The Macmillan Press Ltd., 1988

### pH distributions in spontaneous and isotransplanted rat tumours

F. Kallinowski & P. Vaupel

*Department of Applied Physiology, University of Mainz, Duesbergweg 6, D-6500 Mainz, Federal Republic of Germany.*

**Summary** Spontaneous mammary tumours of the rat with various degrees of malignancy exhibit similar tissue pH distributions. The mean pH (±s.d.) of dysplasia is 7.05±0.20. In benign tumours the mean pH is 6.95±0.19 and in malignant tumours it is 6.94±0.19. In contrast, tumours with the same degree of malignancy but different histologies show different pH distributions. Benign tumours with a higher percentage of fibrous tissue exhibit less acidic pH values than those with larger portions of epithelial cells ($\Delta$pH=0.38 pH units). The pH distribution in the benign tumours is independent of the tumour wet weight up to stages of very advanced growth. In the malignant tumours, a trend towards more acidic pH values is observed as the tumour mass enlarges. However, in tissue areas within a malignant tumour with gross, long-established necrosis the pH distribution is shifted towards more alkaline pH values. The pH distributions in spontaneous rat tumours are not significantly different from those obtained in isotransplanted Yoshida sarcomas (6.87±0.21). In the Yoshida sarcomas, mean pH values do not correlate with tumour size. However, a pH gradient from the rim to the centre of the tumours is found which coincides with the development of small, disseminated necroses in the tumour centre. It is concluded that pathology-related variations of tumour pH may be more important than the mode of tumour origin or the degree of malignancy.

In contrast to normal tissues, in most malignant tumours, an inadequate and non-uniform microcirculation develops with tumour growth (for a review see Vaupel *et al.*, 1981). Concomitantly, typical alterations in the metabolic micromilieu occur characterized by hypoxia (and eventually anoxia), a general deprivation of nutrients and energy sources, and an insufficient removal of metabolic waste products, predominantly lactic acid. Thus, tissue acidosis is a typical feature of the metabolic micromilieu of numerous human and murine tumours (Vaupel *et al.*, 1981; Wike-Hooley *et al.*, 1985). Low tumour pH values can influence the efficacy of various non-surgical tumour therapies, such as irradiation, chemotherapy and hyperthermia (for a review see Wike-Hooley *et al.*, 1984). Therefore, the existence of pathology-related pH variations, for example due to differing histologies or due to varying degrees of malignancy, might be of practical importance. Since conclusive data are not available so far, dysplasias, benign and malignant tumours in the rat are investigated to reveal possible pH differences. Furthermore, this investigation is aimed to clarify whether spontaneous and isotransplanted tumours exhibit different pH distributions. This is important since most of the pH data available have been obtained from isotransplanted murine tumours.

### Materials and methods

### *Animals and tumours*

*Spontaneous tumours* ($n=27$), that grew along the milk line of Sprague–Dawley rats were used throughout the experiments. Tumour-bearing animals (both sexes; 225–640 g), obtained from the breeding colonies of Hoechst AG (Frankfurt/M., FRG), from the Department for Animal Experimentation, University of Frankfurt (Frankfurt/M., FRG) and from the Department of Applied Physiology, University of Mainz (Mainz, FRG) were submitted to the study. Furthermore, pH distributions were measured in isotransplanted *Yoshida sarcomas* ($n=30$). The sarcomas grew s.c. in the hind foot dorsum of SD-rats of both sexes (180–560 g) after inoculation of ascites cells (0.4 ml; ca. $10^4$ cells $\mu l^{-1}$). The Yoshida sarcoma was originally obtained from the German Cancer Research Centre, Heidelberg (FRG), and was serially passaged as ascites tumour in the abdominal cavity of SD-rats (120–180 g). As controls, pH values were measured in the subcutis and in skeletal muscle of 23 healthy Sprague–Dawley rats. All rats were kept in Makrolon cages bedded with dust-free wood granulate (2–3 animals per cage; 12 hourly light/dark cycles). The animals were fed Altromin 1324 diet and obtained drinking water *ad libitum*.

### *Measurements of tissue pH values with miniaturized needle glass electrodes.*

pH measurements were performed with steel-sheathed, miniaturized needle pH electrodes (type MI 408 B, Microelectrodes, Inc., Londonderry, NH, USA; diameter of the sensitive tip: 650 $\mu$m). Electrodes of this type were chosen for their mechanical stability and for comparison with pH values measured in primary tumours in patients (Thistlethwaite *et al.*, 1985). A macro-calomel-electrode served as a reference (type 303, Ingold, Frankfurt/M., FRG). The pH probe was mounted on a micromanipulator (type MM5m with control device STM 3, Maerzhaeuser, Wetzlar, FRG), and the reference electrode was fixed with a standard laboratory stand. The electrodes were connected to a two-channel voltmeter (type 619, Keithley Instruments, Cleveland, Ohio, USA) and the potential difference was recorded on a standard chart recorder (type LS 23, Linseis GmbH, Selb, FRG). Before and after each tissue track, the electrodes were calibrated in 3 different buffer solutions, thermostatized at 34°C (pH 4.02, 6.84, and 9.08, Schott, Hofheim, FRG). This temperature corresponded to the mean temperature of the tissues investigated as measured in a separate series. The electrodes were routinely cleaned overnight in a 5% protease solution (P-4630, Type I, Sigma Chemicals, St. Louis, MO, USA).

In different buffer solutions, the electrodes reached the 95% value in <1 min. The electrode drift was less than 0.02 pH units $h^{-1}$, the calibration reproducibility after use in tissue usually was within 0.04 pH units of the initial value. The electrode signal was linear within a pH range of 4 to 10. The sensitivity was ca. 58 mV/pH unit at 34°C (theoretical Nernst's potential: 60 mV/pH unit at 34°C). In tissue, it usually took <15 min for the signal to reach a stable level after the insertion of the electrode (Figure 1b). The electrode responded to i.v. application of bicarbonate only if gross changes of the acid-base status of the arterial blood were observed. On application of glucose (3 g $kg^{-1}$ i.v.) tumour pH dropped up to 0.4 pH units within 30–60 min while arterial blood glucose levels were elevated up to 20 mM.

### *Experimental protocol*

The animals were anaesthetized with Pentobarbital-Na

Correspondence: F. Kallinowski.
Received 18 May 1987; and in revised form 3 February 1988.

**Figure 1** (**a**) Schematic representation of the position of pH-sensitive electrodes (1, 2) along the longest axis of Yoshida sarcomas s.c. isotransplanted in the hind foot dorsum of SD-rats (shaded area).

(**b**) Original pH recordings in an isotransplanted Yoshida sarcoma with a wet weight of 2.3 g (length: 27 mm; width: 15 mm; height: 10 mm). Two tracks were performed at the same time. At each break in the recordings the electrodes were advanced by 500 $\mu$m. The arrow indicates the time of sampling of arterial blood for the determination of arterial blood gases and hematocrit (MABP = mean arterial blood pressure).

(40 mg $kg^{-1}$ i.p.; Nembutal, Ceva, Paris, France) and anti-coagulated with heparin (350 USP-units $kg^{-1}$ $h^{-1}$; Thrombophob, Nordmark, Uetersen, FRG). A catheter in the left carotid artery allowed the continuous monitoring of the mean arterial blood pressure (Statham pressure transducer, type P 23 ID; Gould blood pressure monitor, type SP 1400, Gould, Oxnard, CA, USA) and the withdrawal of blood for the determination of relevant arterial blood gas parameters using a standard blood gas analyzer ($O_2$- and $CO_2$-partial pressures and pH values; type MT 33, Eschweiler, Kiel, FRG). The arterial haematocrit (Hct) was measured using the Hawksley micromethod. The $O_2$ saturation of the arterial blood was obtained nomographically according to Bork *et al.* (1975). Blood loss due to sampling was adequately replaced with fresh donor blood. The rectal temperature was kept at 37°C by placing the animal on a heating pad.

After careful removal of the overlying skin and subcutaneous tissue, the pH electrode was inserted into the tumour tissue to an initial depth of 250–500 $\mu$m. The reference electrode was placed into the subcutis nearby. The insertion sites were moisturized with 0.9% NaCl-solution (T = 34°C).

In the spontaneous tumours, the electrodes were placed randomly. The number of pH measurements per tumour as well as the diameters along the three major axes are given in Table I. Due to the anatomical localization of the Yoshida sarcomas it was possible to advance the electrodes radially along the longest axis of the tumours avoiding the plane of the metatarsal bones. Here, one electrode track was performed in more proximal parts of the tumours and another one more distally (Figure 1a). The distance between the tracks was 4–8 mm. The mean diameters of tumours (length · width · height) with wet weights ~1.4 g were 23 · 14 · 8 mm and 31 · 20 · 12 mm for tumour sizes ~4 g. Tumour heights were always measured excluding the plane of the metatarsal bones. In the Yoshida sarcomas 40–50 measurements were taken regardless of tumour size. For measurements of subcutis and muscle pH the electrodes were inserted into the tissues and progressively pushed forward. Here, 2–8 pH values were taken per animal.

**Table I** Histologies, tumour wet weight (tww), largest diameter along the major axes (length, width, height), volume fraction of necrosis, number of pH readings (N) and mean tissue pH value (pH)

| *Histology* | *tww (g)* | *Length (mm)* | *Width (mm)* | *Height (mm)* | *Necrosis (%)* | *N* | *pH* |
|---|---|---|---|---|---|---|---|
| (*a*) *Dysplasias* | | | | | | | |
| Ductectasia | 2.0 | 20 | 15 | 13 | | 39 | 7.07 |
| Ductectasia | 2.1 | 21 | 15 | 13 | no | 72 | 7.12 |
| Ductectasia | 7.6 | 40 | 20 | 18 | | 43 | 7.27 |
| Adenosis | 20.0 | 53 | 35 | 21 | | 43 | 6.72 |
| (*b*) *Benign tumours* | | | | | | | |
| Compound tumour | 2.4 | 21 | 17 | 14 | | 58 | 6.92 |
| Fibroadenoma | 7.2 | 39 | 19 | 18 | | 43 | 7.25 |
| Fibroadenoma | 8.0 | 30 | 28 | 18 | | 41 | 6.90 |
| Adenoma | 8.4 | 31 | 29 | 18 | | 88 | 6.85 |
| Fibroadenoma | 8.7 | 31 | 29 | 19 | | 41 | 6.99 |
| Adenoma | 13.0 | 35 | 34 | 21 | | 45 | 6.82 |
| Fibroadenoma | 15.1 | 35 | 35 | 24 | | 62 | 7.02 |
| Compound tumour | 25.0 | 45 | 45 | 24 | | 68 | 6.76 |
| Compound tumour | 27.0 | 50 | 48 | 21 | | 50 | 7.21 |
| Compound tumour | 31.0 | 51 | 46 | 26 | no | 117 | 6.83 |
| Compound tumour | 33.0 | 53 | 51 | 23 | | 52 | 6.73 |
| Compound tumour | 35.0 | 55 | 53 | 23 | | 55 | 6.90 |
| Compound tumour | 42.0 | 58 | 56 | 25 | | 51 | 7.19 |
| Compound tumour | 44.0 | 58 | 58 | 25 | | 53 | 7.26 |
| Compound tumour | 56.0 | 62 | 60 | 28 | | 57 | 6.90 |
| Fibroadenoma | 73.0 | 70 | 65 | 31 | | 50 | 6.98 |
| Fibroadenoma | 101.0 | 72 | 72 | 37 | | 62 | 6.95 |
| Fibroma | 180.0 | 78 | 74 | 59 | | 53 | 6.88 |
| (*c*) *Malignant tumours* | | | | | | | |
| Anaplastic adenocarcinoma | 7.0 | 34 | 21 | 18 | 5 | 34 | 7.16 |
| Squamous cell carcinoma | 10.8 | 34 | 34 | 18 | ca. 70 | 81 | 7.04 |
| Anaplastic compound tumour | 17.0 | 36 | 32 | 28 | 5 | 76 | 6.97 |
| Anaplastic adenocarcinoma | 30.5 | 42 | 40 | 35 | ca. 35 | 48 | 6.89 |
| Anaplastic adenocarcinoma | 72.5 | 68 | 67 | 30 | 5 | 77 | 6.72 |

*Histological investigations*

At the end of the experiments, all tumours were excised, weighed and examined by standard histological techniques in order to get a first estimate of the vascular pattern as well as of the volume fraction and distribution of necroses within the tumours. For the assessment of the vascular pattern, blood conducting channels filled with erythrocytes within the tumour tissue were evaluated. Necrosis was defined as tumour areas with loss of clearly defined cell membranes with and without pycnotic nuclei. Additionally, the spontaneous tumours were classified according to Komitowski *et al.* (1982). These authors suggested a classification of rat mammary tumours based on more than 2,500 tumours of the mammary gland obtained from different rat strains. At least six different sections were analyzed from each individual tumour.

*Statistical evaluation*

In order to gain an insight into the intra-tumour pH distribution, the measured pH values were grouped into relative frequency histograms (pH-histograms; class width: 0.1 pH units). For each tumour, mean and median pH values as well as the modal class were determined. In order to evaluate statistically significant group differences, the Kruskal–Wallis-test as well as the U-tests for paired and unpaired samples were used. For these calculations, the tumours were represented by their median pH values. Values reported are means ±s.d. unless otherwise stated.

### Results

The pH values obtained during normal acid-base status (Table II) in spontaneous and isotransplanted rat tumours were lower than those measured in the normal tissues at the site of growth (*skeletal muscle* and *subcutis*, $2P<0.001$). The mean pH value in the thigh musculature of SD-rats was 7.26±0.12, and in the subcutis 7.32±0.12.

Four out of the 27 *spontaneous rat tumours* were classified as *dysplasias* (Table I). The mean pH value in these dysplasias was 7.05±0.20, i.e., lower than that of the normal tissues. No necrosis was found in the dysplasias. In most dysplasias, heavy ectasia of ducts was seen leading to swelling, multiple cysts and transformation of the mammary gland into a spongy mass. Here, very few vessels were found trailing in the connective tissue which supported the grossly dilated ducts. In the case of the adenosis, an increase in the size, complexity and number of the mammary lobules was noted. In some areas, only a few vessels were observed within the stroma surrounding lobules up to 1.5 mm in diameter. In other areas, many vessels filled with erythrocytes were present with a mean intervascular distance around 100 μm.

In 18 *benign rat mammary tumours* investigated (see Table I), the mean tissue pH value was 6.95±0.19 (Figure 2). This pH value did not differ significantly from that found in dysplasias. Here again, no necrosis was detectable. In these tumours, a wide variety of histological features was present ranging from compactly arranged tubular structures surrounded by delicate connective tissue fibers (adenomas) to a complex morphology with tubular, pseudopapillary and highly cellular formations (compound tumours) to tubules in abundant fibrous stroma (fibroadenomas) and fibrous tissue only (fibromas). Similarly, the vascular pattern was very different in the various histological types. In more epithelial tumours both rarefaction of vessels and hypervascularization was observed. As a rule, very few vessels were found in more fibrous tumour areas. No clear correlation between the mean tissue pH and the tumour size was found in the benign tumours up to very advanced growth stages. Considering benign tumours with different histologies separately different pH distributions were found ($2P<0.01$, Figure 3). Average pH values of 7.22±0.18 were obtained in compound tumours with a higher percentage of fibrous tissue. In fibroadenomas, mean pH values around 7.02±0.22 were observed. In compound tumours with larger portions of epithelial tissue a mean tissue pH of 6.84±0.19 was measured. Similar mean pH values were found in the two adenomas investigated (6.85 and 6.82, resp.).

Out of 27 spontaneous tumours, 5 tumours were classified as malignant (Table I). The mean tissue pH value in these *malignant tumours* was 6.94±0.19, i.e., it was similar to that in dysplasias or benign tumours. In two tumours, large necrotic areas were found (1 squamous cell carcinoma, 1 anaplastic adenocarcinoma). The other tumours exhibited only minor amounts of necrosis (2 anaplastic adenocarcinomas, 1 anaplastic compound tumour). The volume fraction of necrosis present did not correlate with the tumour size. These tumours were generally found to be highly cellular with various amounts of fibrous tissue. Both hypo- and hypervascularized tumour areas were present, intercapillary distances varying widely (<100 to >500 μm). In the malignant tumours a trend towards more acidic pH values was found as the tumours increased in size (Figure 4). However, in areas with gross, presumably long-established necrosis, a pH shift towards more alkaline values occurred. This was readily observed in the squamous cell carcinoma. One electrode track was measured in a large and obviously long-existing central necrosis whereas a second track was performed in adjacent vital tissue (Figure 5a). From the results obtained (Figure 5b) it is obvious that pH values between 7.15 and 7.30 were found in necrotic tissue, the pH in vital areas ranging between 6.63 and 7.08. However, the mean pH value even of necrotic tumour areas is still lower than that of the arterial blood.

In the *isotransplanted Yoshida sarcomas*, the mean pH value was 6.87±0.21 (Figure 2) being not significantly different from that in dysplasias and in spontaneously growing benign or malignant tumours. In the sarcomas, no clear-cut relationship was found between the tumour growth stage and the amount of necrosis present. Disseminated areas of necrosis were already obvious in small tumours. However, confluent necroses developed only rarely even at advanced growth stages. Nevertheless, the number of small necroses was higher in the tumour centre than in more peripheral tissue layers. The tumour vasculature seemingly arose from preexisting vessels in the subcutis and the tumour base, the number of erythrocyte-filled channels within the tissue being greater in the periphery than in the tumour centre. Here again, highly vascularised tumour areas were close to tissue regions with almost no vascularization. Considering tumour pH as a function of tumour weight, the pH distributions were not significantly different in small (mean tumour wet

**Table II** $O_2$ partial pressure ($pO_2$), $CO_2$ partial pressure ($pCO_2$), pH, oxygen saturation ($So_2$), haematocrit (hct) values of arterial blood and the mean arterial blood pressures (MABP) of Sprague–Dawley rats with spontaneous mammary tumours or isotransplanted Yoshida sarcomas. Values are means±s.d.

| *Tumour type* | $pO_2$ *(mmHg)* | $pCO_2$ *(mmHg)* | *pH* | $So_2$ *(sat. %)* | *hct (v/v)* | *MABP (mmHg)* |
|---|---|---|---|---|---|---|
| Spontaneous mammary tumours | 92± 9 | 37±5 | 7.38±0.04 | 97±4 | 0.44±0.05 | 119±18 |
| Yoshida sarcomas | 80±10 | 42±6 | 7.37±0.04 | 95±2 | 0.39±0.06 | 121±17 |

**Figure 2** pH histograms for spontaneous benign rat tumours and isotransplanted Yoshida sarcomas. The broken lines indicate the respective mean value ($N$ = number of tumours investigated, $n$ = number of pH values measured).

**Figure 3** pH histograms for fibrous compound tumours (**a**), for fibroadenomas (**b**) and for compound tumours with a higher portion of epithelial tissue (**c**). The broken lines indicate the mean tumour pH values ($N$ = number of animals investigated, $n$ = number of pH measurements taken).

**Figure 4** Mean pH values (±s.d.) of malignant rat tumours with different wet weights. The broken line indicates the trend.

**Figure 5** (**a**) Schematic representation of the position of the pH-sensitive electrodes (1, 2) in a spontaneous squamous cell carcinoma of the rat. The tumour was located at the abdominal wall and was almost circular in shape (length: 34 mm; width: 34 mm; height: 18 mm; wet weight: 10.8 g).

(**b**) Tissue pH values along two different electrode tracks in a spontaneous squamous cell carcinoma of the rat. The upper track (circles) was measured in a gross central necrosis whereas the lower track (dots) was obtained in adjacent vital tissue. Insertion depth relates to the first data point which is approx. 250–500 $\mu$m inside the tumours.

weight, tww: 1.5±0.4 g; mean pH: 6.85±0.17), in medium size (tww: 2.4±0.4 g; mean pH: 6.89±0.24), and in larger Yoshida sarcomas (mean tww: 4.0±0.7 g; mean pH: 6.86±0.21). Since it was possible to advance the pH electrodes on radial tracks through the Yoshida sarcomas due to their anatomical localization (Figure 1a), mean pH gradients from the outer layers to the centre could be evaluated in these tumours. The pH distribution shifted to more acidic values as the electrodes were advanced from the outer rim to the more central layers ($2P<0.001$). The mean pH value in the outer layer (0.5–3.5 mm) of the Yoshida sarcomas investigated was 6.95±0.18 (upper panel in Figure 6), in the intermediate layer (4–7 mm) 6.84±0.16 (central panel in Figure 6), and in central portions of the tumours 6.78±0.18 (lower panel in Figure 6). In the spontaneous tumours, no such pattern was found probably due to a random positioning of the electrodes. However, it has to be mentioned that in the Yoshida sarcomas as well as in the other tumours marked inter- and intra-tumour pH variations have been obtained (Figure 7). In individual tumours, steep pH gradients, a pH decrease followed by a subsequent pH increase and vice versa have all been found along a measured electrode track. pH values measured in more proximal parts of the tumours (Figure 1a) were not significantly different from those in more distal parts.

### Discussion

### *Methods*

Due to the *electrode size* the tissue pH measured is a mixture of intravascular, interstitial and intracellular pH values. Since the interstitial space is large in malignant tumours (Gullino, 1975; Vaupel & Hammersen, 1983) the value obtained is determined, to a large extent, by the interstitial pH value. The amount of tissue damage due to the measurement should increase at larger tip diameters leading to

**Figure 6** Frequency distributions of pH values measured in the outer layers (0.5–3.5 mm, **a**), in intermediate zones (4–7 mm, **b**) and in central areas (7.5–10.5 mm, **c**) of 30 isotransplanted Yoshida sarcomas. The broken lines indicate the respective mean pH values ($n$ = number of pH values measured).

**Figure 7** pH profiles in two isotransplanted Yoshida sarcomas (tumour # NT 18 and # NT 19; tww = tumour wet weight). The insertion depth relates to the first data point which is approx. 250–500 μm inside the tumours.

erroneous pH determinations. However, there is little experimental evidence to support this argument. Using electrodes with tip diameters up to 2.1 mm inserted into solid rat tumours, Wike-Hooley *et al.* (1985) have found surprisingly little disruption of blood vessels and haemorrhages around the tip of the electrode. In the present study, the electrode track has rarely been found in isotransplanted Yoshida sarcomas on multiple sections. Further evidence comes from experiments performed by Song *et al.* (1980). These authors found pH values around 7.05 in SCK mammary adenocarcinomas using an electrode with a tip diameter of 0.8 mm. In the same tumour type, Rhee *et al.* (1984) obtained pH distributions ranging from 6.60 to 7.38 (mean: 6.96) with a much smaller electrode (tip diameter: 50–80 μm). Compiling all pH data obtained in malignant tumours of human beings or rodents up to now, similar pH values were found with large and small electrodes. Using tip diameters below 10 μm, the pH values range between 6.59 and 7.15. Considering electrodes with tip diameters between 1 and 5 mm, mean pH values between 6.74 and 7.29 were found (for a review see Wike-Hooley *et al.*, 1984). To the best of our knowledge, no systematic studies were performed so far correlating measured pH values with the size of the electrode tips. Thus, it has to be concluded that very fine tip diameters are necessary for the detailed investigation of pH distributions in tumour microareas whereas reasonable estimates of tumour tissue pH can be obtained with larger electrode sizes.

The interior of tumour cells is generally found to be electronegative as compared to the extracellular space. The *transmembrane potential* varies between −9 and −57 mV with most values found in the range of −10 to −25 mV (Bernhardt & Pauly, 1967; Borle & Loveday, 1968; Hause *et al.*, 1970; Timmermann & von Buttlar, 1978; Walliser & Redmann, 1978; Redmann, 1981; Acker *et al.*, 1983; Gstrein *et al.*, 1987). Since the possibility of proper intracellular pH measurements can be ruled out due to the electrode size, the membrane potential of tumour cells is unlikely to influence significantly the pH values measured in the present study. Biological *electropotentials* vary widely. In general, tumour tissue is about 10 to 15 mV more electronegative than normal tissue (Schauble & Habal, 1970). In order to minimize a possible influence of different electropotentials between normal and tumour tissue the reference electrode was always inserted into the same place with respect to the measuring electrodes (~3 cm apart).

Another common source of error in pH measurements is the use of a porous ceramic type of liquid junction in the reference half-cell which can produce substantial *liquid junction potentials* varying with the ionic composition of the solution under test (Illingworth, 1981). This was tested using buffers with different ionic composition. It was found that the error introduced by this way in our system is about 0.02 pH units/10-fold salt concentration difference between standard and test solution. As an approach to rule out this possible artifact buffers with physiological ionic strength were used.

*Results*

Spontaneous mammary tumours of rats resemble human breast tumours in their hormone sensitivity and their histology (Young & Hallowes, 1973). In these rat tumours, pH distributions can be measured, whereas in patient tumours ethical and practical reasons permit at the best only a few pH determinations which may not be adequate considering the pronounced intra-tumour pH variations reported previously (Vaupel *et al.*, 1981). Thus spontaneous mammary tumours of the rat may be used as a first approach to evaluate possible variations of the pH distributions related to varying degrees of malignancy. To the best of our knowledge, comparative pH studies similar to those presented here have not been performed before. This may partly be due to the low incidence rate or the long latency period of spontaneous tumours (for a review see Young & Hallowes, 1973). In the only other study comparing pH values of malignant and benign lesions of patients done by Meyer *et al.* (1948), severe artifacts cannot be excluded since the pH determinations were made in excised tissues, i.e., *ex vivo*. The results obtained in the present study were compared with those found in isotransplanted Yoshida sarcomas. The Yoshida sarcoma was previously used for pH measurements as well as for susceptibility studies to anticancer drugs and hyperthermia (Dickson & Suzangar, 1974; Dickson & Ellis, 1976; Schmaehl, 1981; Dickson & Calderwood, 1979, 1983).

The pH values obtained in skeletal muscle and in the skin of rats are within the range of values reported earlier (range: 7.20–7.59; Voegtlin *et al.*, 1935; Tagashira *et al.*, 1953; Eden *et al.*, 1955; Kahler & Moore, 1962; Gullino *et al.*, 1965; Rauen *et al.*, 1968; Gebert & Friedman, 1973; Dickson & Calderwood, 1979, 1983; van den Berg *et al.*, 1982; Hinsull *et al.*, 1984; Jain *et al.*, 1984). Since mean pH values in the skeletal muscle of rats higher than those of the arterial blood were reported (Voegtlin *et al.*, 1935; Kahler & Moore, 1962; Rauen *et al.*, 1968; van den Berg *et al.*, 1982), comparative measurements were performed, in which the techniques used for pH measurements by van den Berg *et al.* (1982) and our group were applied to the same animal. In contrast to the values by van den Berg *et al.* (1982) and in agreement with the values reported here, the mean pH value in the rat subcutis was found to be 7.35 with our electrode and 7.28 using the Philips electrode employed by the Rotterdam Radio-Therapeutic Institute (Wike-Hooley *et al.*, 1985). Possible reasons for elevated pH values in normal tissues include temperature differences between calibration vessel and tissue and $CO_2$ losses from the tissues during the measurement.

Compared to the values in normal tissues, the pH distributions in the tumours investigated are generally shifted to more acidic values. This finding is in good agreement with data measured in rat tumours with various methods including glass and antimony electrodes, collection of interstitial fluid, $^{31}$phosphorus magnetic resonance spectroscopy and distribution of weak acids and bases (range of mean pH: 6.59–7.25; Voegtlin *et al.*, 1935; Kahler & Robertson, 1943; Tagashira *et al.*, 1953, 1954; Eden *et al.*, 1955; Scheid & Kunze, 1962; Kahler & Moore, 1962; Gullino *et al.*, 1965; Rauen *et al.*, 1968; von Ardenne & Reitnauer, 1976, 1979; Dickson & Calderwood, 1979; Song *et al.*, 1980; Mueller-Klieser *et al.*, 1981; Busse *et al.*, 1981; van den Berg *et al.*, 1982; Jaehde *et al.*, 1982; Dickson & Calderwood, 1983; Hinsull *et al.*, 1984; Jain *et al.*, 1984; Koeze *et al.*, 1984; Arnold *et al.*, 1985; Osinsky *et al.*, 1987).

In the present study, the pH values in dysplasias, benign and malignant rat tumours were not significantly different. However, on further analysis, the various histological types have to be considered separately. In the case of the *dysplasias*, the ductectasias showed considerably higher pH values than the adenosis investigated, i.e., the tumour with the higher percentage of epithelial tissue is more acidic. Furthermore, the ductectasias consist mainly of distended ducts filled with protein-rich material with a high buffering capacity which may well prevent any pronounced pH drop.

Considering the *benign tumours*, the majority have both fibrous and epithelial portions (compound tumours and fibroadenomas). Here again, tumours with a larger portion of epithelial cells have more acidic pH values than those with a higher content of fibrous tissue. It may be speculated that the former tumours may have a higher glycolytic rate and thus may exhibit more acidic pH values. Another possibility would be that a higher proliferation rate of these tumours would lead to a high interstitial pressure followed by vascular compression. Unfortunately, no data are available on the growth rate of these tumours. However, in a study on various isotransplanted rat tumours no dependency of the mean tumour pH and the overall growth rate was found (Eden *et al.*, 1955).

In the *malignant tumours*, the pH shift to more acidic values is most probably due to an impaired microcirculation with severe restrictions of convective and diffusive transport in combination with a high glycolytic rate of the tumour tissue both in the presence and absence of oxygen. The reduction of blood flow per unit mass found for many rodent tumours (Vaupel, 1974; Gullino, 1975) leads to a restricted oxygen supply and thus to the development of hypoxic and anoxic areas in malignant tumours (Vaupel *et al.*, 1981). Since the diffusion distance for glucose seems to be larger than that for $O_2$ in tumour tissue (Kallinowski & Vaupel, 1986), hypoxic tumour cells can still cleave glucose to lactic acid for energy production, thus causing tumour acidosis. In recent years, evidence has come from tissue cultures that under *in vitro* conditions lactic acid is derived from glutamine rather than from glucose (for a review see Eigenbrodt *et al.*, 1985). However, since oxygen is essential for the reoxidation of reduced coenzymes and reduced cytochromes necessary for the breakdown of glutamine to lactic acid, only well oxygenated cells can convert glutamine to lactic acid (Kallinowski *et al.*, 1987).

The pH distributions in spontaneous rat tumours were not significantly different from those found in *isotransplanted tumours* of the rat. This finding is in agreement with the results of Kahler & Robertson (1943), who investigated spontaneous and isotransplanted hepatomas. Thus, considering all the evidence available so far, it has to be concluded, that pH values 0.2 to 0.6 pH units lower than those of normal tissues at the site of growth are usually found in rat tumours regardless of the mode of origin or the degree of malignancy.

Many tumours exhibit a 'peripheral' blood supply with an increasing rarefaction of the vasculature going from outer to inner tumour regions (Scheid, 1961; Mueller-Klieser *et al.*, 1980). This explains the finding that, as a rule, a mean pH gradient from the outer layers to more central areas has been found in the Yoshida sarcomas as well as in other tumours (Jaehde & Rajewsky, 1982; Jaehde *et al.*, 1982; Dickson & Calderwood, 1983; Koeze *et al.*, 1984; Rhee *et al.*, 1984). Using one electrode or two electrodes simultaneously gave the same results. Thus, the pH decrease with increasing insertion depth is not due to a pressure artifact caused by pushing one electrode against the other. Data from Yoshida sarcomas of different sizes were compiled since the mean pH values decreased with increasing insertion depth in all weight groups investigated. However, in individual tumours, quite different patterns can be found due to the non-homogeneous distribution of blood flow, oxygen and substrates within the tissue.

The pH distributions in the spontaneous benign tumours and in the Yoshida sarcomas do not shift to more acidic values as the tumours increase in size. Similar findings have been reported earlier by Vaupel *et al.* (1981). On the other hand a pH decrease with increasing size has been found (Kahler & Moore, 1962; Jaehde *et al.*, 1982; Jain *et al.*, 1984;

Thistlethwaite *et al.*, 1985; Rhee *et al.*, 1985). A similar trend was observed in the spontaneous malignant rat tumours. A continuous monitoring of tumour pH during growth shows, however, that initially a pH drop occurs in small tumours, followed by a pH increase at advanced growth stages (Hinsull *et al.*, 1984). These controversial results may be explained by the fact that with increasing tumour size, the impaired tumour blood flow leads to severe restrictions not only of the oxygen supply but also of the glucose delivery (Gullino *et al.*, 1967; Vaupel, 1974). Additionally, regressive changes and necroses may develop as tumours increase in size. During the development of necrosis, hydrolysis of ATP occurs resulting in a pronounced pH drop (Hochachka & Mommsen, 1983). Such a mechanism could also be responsible for the pH drop from the outer rim to the centre of the Yoshida sarcomas since small, disseminated necroses were more frequent in the centre than in the rim of these tumours. Within the necrotic areas, glycolysis and $CO_2$ production cease and proton-binding structures are exposed alleviating the acidosis of the tissue in longstanding necrosis (Vaupel *et al.*, 1981).

The tumour vasculature determines, to a large extent, the nutritive tumour blood flow. From the evidence available so far, it has to be concluded that the vascular morphology may be characteristic but not unique for a specific tumour. Furthermore, the histological type of a tumour and the degree of malignancy certainly modulate and may even dictate the vascular pattern (for reviews see Peterson, 1979; Vaupel & Gabbert, 1986). Variations of tumour vasculature with subsequent differences of the tumour micromilieu (hypoxia, acidosis) are therapeutically highly relevant both in experimental rodent and spontaneous human tumours (Cole *et al.*, 1983; Révész & Siracka, 1984). Thus, pathology-related changes of the tumour micromilieu need to be further evaluated.

We express our deepest thanks to Dr Weisse from the Department of Animal Pathology, Boehringer-Ingelheim GmbH (Ingelheim, FRG) who performed the histological classifications of the spontaneous tumours. Furthermore, we wish to thank M. Kluge for technical assistance during some of the pH measurements.

### References

ACKER, H., CARLSSON, J. & STALNACKE, C.G. (1983). Electrophysiological measurements in cultured cellular spheroids. *Acta Path. Microbiol. Immunol. Scand. Sect. A*, **91**, 151.

ARDENNE, M. VON & REITNAUER, P.G. (1976). Verstärkung der mit Glukoseinfusion Herzielbaren Tumorübersäuerung in vivo durch NAD. *Arch. Geschwulstforsch.*, **46**, 197.

ARDENNE, M. VON & REITNAUER, P.G. (1979). Verstärkung der mit Glukoseinfusion erzielbaren Tumorübersäuerung durch lokale Hyperthermie. *Res. Exp. Med.*, **175**, 7.

ARNOLD, J.B., JUNCK, L. & ROTTENBERG, D.A. (1985). *In vivo* measurement of regional brain and tumor pH using $^{14}C$ dimethyloxazolidinedione and quantitative autoradiography. *J. Cereb. Blood Flow Metabol.*, **5**, 369.

BERG, A.P. VAN DEN, WIKE-HOOLEY, J.L., BERG-BLOK, A.E. VAN DEN, ZEE, J. VAN DER & REINHOLD, H.S. (1982). Tumour pH in human mammary carcinoma. *Eur. J. Cancer Clin. Oncol.*, **18**, 457.

BERNHARDT, J. & PAULY, H. (1967). Das Membranpotential von Ehrlich-Aszitestumorzellen. *Biophysik*, **4**, 101.

BORK, R., VAUPEL, P., GUENTHER, H. & THEWS, G. (1975). Atemgas-pH-Nomogramme für das Rattenblut bei 37°C. *Anaesthesist*, **24**, 84.

BORLE, A.B. & LOVEDAY, J. (1968). Effects of temperature, potassium, and calcium on the electrical potential difference in HeLa cells. *Cancer Res.*, **28**, 2401.

BUSSE, J., MUELLER-KLIESER, W. & VAUPEL, P. (1981). Intratumor pH distribution – a function of tumor growth stage? *Pfluegers Arch.*, **389**, R 55.

COLE, M.A., CRAWFORD, D.W., WARNER, N.E. & PUFFER, H.W. (1983). Correlation of regional disease and *in vivo* $pO_2$ in rat mammary adenocarcinoma. *Amer. J. Pathol.*, **112**, 61.

DICKSON, J.A. & SUZANGAR, M. (1974). *In vitro–in vivo* studies on the susceptibility of the solid Yoshida sarcoma to drugs and hyperthermia (42°C). *Cancer Res.*, **34**, 1263.

DICKSON, J.A. & ELLIS, H.A. (1976). The influence of tumor volume and the degree of heating on the response of the solid Yoshida sarcoma to hyperthermia (40–42°). *Cancer Res.*, **38**, 1188.

DICKSON, J.A. & CALDERWOOD, S.K. (1979). Effects of hyperglycemia and hyperthermia on the pH, glycolysis, and respiration of the Yoshida sarcoma *in vivo*. *J. Natl Cancer Inst.*, **63**, 1371.

DICKSON, J.A. & CALDERWOOD, S.K. (1983). Thermosensitivity of neoplastic tissue *in vivo*. In *Hyperthermia in Cancer Therapy*, Storm, F.K. (ed) p. 63 Hall Publishers: Boston.

EDEN, M., HAINES, B. & KAHLER, H. (1955). The pH of rat tumors measured *in vivo*. *J. Natl Cancer Inst.*, **16**, 541.

EIGENBRODT, E., FISTER, P. & REINACHER, M. (1985). New perspectives on carbohydrate metabolism in tumor cells. In *Regulation of Carbohydrate Metabolism* (Vol. II). R. Beitner (ed) p. 141. CRC Press: Boca Raton.

GEBERT, G. & FRIEDMAN, S.M. (1973). An implantable glass electrode used for pH measurement in working skeletal muscle. *J. Appl. Physiol.*, **34**, 122.

GSTREIN, E., PAULMICHL, M. & LANG, F. (1987). Electrical properties of Ehrlich ascites tumor cells. *Pfluegers Arch.*, **408**, 432.

GULLINO, P.M. (1975). Extracellular compartments of solid tumors. In *Cancer* (Vol 3). Becker, F.F. (ed) p. 327. Plenum Press: New York.

GULLINO, P.M., GRANTHAM, F.H., SMITH, S.H. & HAGGERTY, A.C. (1965). Modifications of the acid-base status of the internal milieu of tumors. *J. Natl Cancer Inst.*, **34**, 857.

GULLINO, P.M., GRANTHAM, F.H. & COURTNEY, A.H. (1967). Glucose consumption by transplanted tumors *in vivo*. *Cancer Res.*, **27**, 1031.

HAUSE, L.L., PATTILLO, R.A., SANCES, A. & MATTINGLY, R.F. (1970). Cell surface coatings and membrane potentials of malignant and nonmalignant cells. *Science*, **169**, 601.

HINSULL, S.M., COLSON, R.H., FRANKLIN, A., WATSON, B.W. & BELLAMY, D. (1984). Determination of extracellular pH and tissue temperature in transplantable rat tumors by use of inductive loop telemetry. *J. Natl Cancer Inst.*, **73**, 463.

HOCHACHKA, P.W. & MOMMSEN, T.P. (1983). Protons and anaerobiosis. *Science*, **219**, 1391.

ILLINGWORTH, J.A. (1981). A common source of error in pH measurements. *Biochem. J.*, **195**, 259.

JAIN, R.K., SHAH, S.A. & FINNEY, P.L. (1984). Continuous noninvasive monitoring of pH and temperature in rat Walker 256 carcinoma during normoglycemia and hyperglycemia. *J. Natl Cancer Inst.*, **73**, 429.

JAEHDE, E. & RAJEWSKY, M.F. (1982). Tumor-selective modification of cellular microenvironment *in vivo*: effect of glucose infusion on the pH in normal and malignant rat tissues. *Cancer Res.*, **42**, 1505.

JAEHDE, E., RAJEWSKY, M.F. & BAUMGAERTL, H. (1982). pH distribution in transplanted neural tumors and normal tissues of BDIX rats as measured with pH microelectrodes. *Cancer Res.*, **42**, 1498.

KAHLER, H. & MOORE, B. (1962). pH of rat tumors and some comparison with the lissamine-green circulation test. *J. Natl Cancer Inst.*, **28**, 561.

KAHLER, H. & ROBERTSON, W.V.B. (1943). Hydrogen-ion concentration of normal liver and hepatic tumors. *J. Natl Cancer Inst.*, **3**, 495.

KALLINOWSKI, F. & VAUPEL, P. (1986). Concurrent measurements of $O_2$ partial pressure and pH values in human mammary carcinoma xenotransplants. *Adv. Exp. Med. Biol.*, **200**, 609.

KALLINOWSKI, F., RUNKEL, S., FORTMEYER, H.P., FOERSTER, H. & VAUPEL, P. (1987). L-glutamine: a major substrate for tumor cells *in vivo*? *J. Cancer Res. Clin. Oncol.*, **113**, 209.

KOEZE, T.H., LANTOS, P.L., ILES, R.A. & GORDON, R.E. (1984). *In vivo* nuclear magnetic resonance spectroscopy of a transplanted brain tumor. *Br. J. Cancer*, **49**, 357.

KOMITOWSKI, D., SASS, B. & LAUB, W. (1982). Rat mammary tumor classification: notes on comparative aspects. *J. Natl Cancer Inst.*, **68**, 147.

MEYER, K.A., KAMMERLING, E.M., AMTMANN, L., KOLLER, M. & HOFFMANN, S.J. (1948). pH studies in malignant tissues in human beings. *Cancer Res.*, **8**, 513.

MUELLER-KLIESER, W., BUSSE, J. & VAUPEL, P. (1981). Tissue pH-distribution within malignant tumors as measured with antimony microelectrodes. *Adv. Physiol. Sci.*, **25**, 253.

MUELLER-KLIESER, W., VAUPEL, P., MANZ, R. & GRUNEWALD, W.A. (1980). Intracapillary oxyhemoglobin saturation in malignant tumours with central or peripheral blood supply. *Eur. J. Cancer*, **16**, 195.

OSINSKY, S., BUBNOVSKAJA, L. & SERGIENKO, T. (1987). Tumour pH under induced hyperglycemia and efficacy of chemotherapy. *Anticancer Res.*, **7**, 199.

PETERSON, H.I. (1979). Tumor blood circulation: angiogenesis, vascular morphology and blood flow of experimental and human tumors. CRC Press: Boca Raton.

RAUEN, H.M., FRIEDRICH, M. & NORPOTH, K. (1968). Die Beziehung zwischen Milchsäurekonzentration und Gewebs-pH beim DS-Carcinosarkom der Ratte. *Z. Naturforsch.*, **23b**, 1018.

REDMANN, K. (1981). Electrophysiologischer *in vitro*-Nachweis einer individuellen Ouabain-Empfindlichkeit menschlicher Ovarialtumoren. *Acta Biol. Med. Germ.*, **40**, 153.

RÉVÉSZ, L. & SIRACKA, E. (1984). Tumor vascularization, hypoxia, staging of tumors and radiocurability. *Strahlentherapie*, **160**, 658.

RHEE, J.G., KIM, T.H., LEVITT, S.H. & SONG, C.W. (1984). Changes in acidity of mouse tumor by hyperthermia. *Int. J. Radiat. Oncol. Biol. Phys.*, **10**, 393.

SCHAUBLE, M.K. & HABAL, M.B. (1970). Electropotentials of surgical specimen. *Arch. Pathol.*, **90**, 411.

SCHEID, P. (1961). Funktionale Besonderheiten der Mikrozirkulation im Karzinom. *Bibl. Anat.*, **1**, 327.

SCHEID, P. & KUNZE, P. (1962). Continuous vital pH-measurement in animal tumours under (additional) metabolic stress. *Acta Univers. Inst. Cancer*, **18**, 256.

SCHMAEHL, D. (1981). Maligne Tumoren. Entstehung – Wachstum – Chemotherapie. 3rd edition, *Editio Cantor*, Aulendorf.

SONG, C.W., KANG, M.S., RHEE, J.G. & LEVITT, S.H. (1980). The effect of hyperthermia on vascular function, pH, and cell survival. *Radiology*, **137**: 795.

TAGASHIRA, Y., YASUHIRA, K., MATSUO, H. & AMANO, S. (1953). Continual pH measuring by means of inserted microglass electrode in living normal and tumor tissues (1st report). *Gann*, **44**, 63.

TAGASHIRA, Y., TAKEDA, S., KAWANO, K. & AMANO, S. (1954). Continuous pH measuring by means of microglass electrode inserted in living normal and tumor tissues (2nd report), with an additional report on interaction of SH-group of animal protein with carcinogenic agent in the carcinogenetic mechanism. *Gann*, **45**, 99.

THISTLETHWAITE, A.J., LEEPER, D.B., MOYLAN, D.J. & NERLINGER, R.E. (1985). pH distribution in human tumors. *Int. J. Radiat. Oncol. Biol. Phys.*, **11**, 1647.

TIMMERMANN, J. & BUTTLAR, M. VON (1978). Membranpotentialuntersuchungen an menschlichen Epithelkarzinomzellen unter ionisierender Bestrahlung. *Strahlentherapie*, **154**, 700.

VAUPEL, P. (1974). Atemgaswechsel and Glukosestoffwechsel von Implantationstumoren (DS-Carcinosarkom) *in vivo*. *Funktionsanalyse biolog, Systeme*, **1**, 1.

VAUPEL, P. & HAMMERSEN, F. (1983). Mikrozirkulation in malignen Tumoren. Karger: Basel, München, Paris, London, New York, Tokyo, Sydney.

VAUPEL, P. & GABBERT, H. (1986). Evidence for and against a tumor type-specific vascularity. *Strahlentherapie Onkol.*, **162**, 633.

VAUPEL, P., FRINAK, S. & BICHER, H.I. (1981). Heterogeneous oxygen partial pressure and pH distribution in C3H mouse mammary adenocarcinoma. *Cancer Res.*, **41**, 2008.

VOEGTLIN, C., FITCH, R.H., KAHLER, H. & THOMPSON, J.W. (1935). Experimental studies on cancer. I. The influence of the parenteral administration of certain sugars on the pH of malignant tumors. *Natl Inst. Health Bull.*, **164**, 1.

WALLISER, S. & REDMANN, K. (1978). Effect of 5-fluoroacil and thymidine on the transmembrane potential and ζ-potential of HeLa cells. *Cancer Res.*, **38**, 3555.

WIKE-HOOLEY, J.L., HAVEMAN, J. & REINHOLD, H.S. (1984). The relevance of tumour pH to the treatment of malignant disease. *Radiother. Oncol*, **2**, 343.

WIKE-HOOLEY, J.L., BERG, A.P. VAN DEN, ZEE, J. VAN DER REINHOLD, H.S. (1985). Human tumor pH and its variations. *Europ. J. Cancer Clin. Oncol.*, **21**, 785.

YOUNG, S. & HALLOWES, R.C. (1973). Tumors of the mammary gland. In *Pathology of Tumours in Laboratory Animals*, Vol. 1/1. Turusov, V.S. (ed) p. 31, IARC 5: Lyon.